# Mycelial Production and Mycorrhizal Establishment of *Terfezia arenaria* and *Tirmania pinoyi* on *Helianthemum canariense*: A Morphological and Molecular Study

**DOI:** 10.3390/microorganisms14102394

**Published:** 2026-10-20

**Authors:** Kaddouri Hicham, Tounsi Abdessamad, Khassali Hamza, Ouahmane Lahcen

**Affiliations:** 1 Unit of Microbial Biotechnologies, Agrosciences, and Environment (BIOMAGE)-CNRST Labeled Research Unit N°4, Laboratory of Water Sciences, Microbial Biotechnologies, and Natural Resources Sustainability (AQUABIOTECH), Faculty of Sciences-Semlalia, Cadi Ayyad University, P.O. Box 2390, Marrakesh 40000, Morocco; hicham.kaddouri@usms.ma; 2 Environmental, Ecological and Agro-Industrial Engineering Laboratory, Faculty of Sciences and Techniques, University Sultan Moulay Slimane, Avenue Mohamed V. BP 591, Beni Mellal 23000, Morocco; abdessamad.tounsi@usms.ac.ma; 3 PHIM, Plant Health Institute of Montpellier, Montpellier University, 34398 Montpellier, France; hamza.khassali@gmail.com

**Keywords:** desert truffles, ectomycorrhizal–endomycorrhizal association, Ascomycota, edible fungi, host plants

## Abstract

Desert truffles are hypogeous edible fungi of ecological and economic importance in arid and semi-arid ecosystems, where they establish mycorrhizal associations with *Helianthemum* species. This study evaluated the effects of pH, culture medium, and fungal species on the apical growth and mycelial biomass production of *Terfezia arenaria* and *Tirmania pinoyi* under controlled conditions. Fungal identification was confirmed by sequencing the ITS region. Both species were cultured on Potato Dextrose Agar and Malt Extract Agar at four pH levels (4.55, 5.6, 6.0, and 6.5) at 28 °C. Mycelial biomass was further evaluated at pH 6.0 and 6.5, and the resulting mycelial suspensions (100 mL) were used to inoculate *Helianthemum canariense* seedlings under controlled conditions. The resulting suspension was applied directly to the root system of young seedlings at a rate of 20 mL per plant. The results revealed highly significant effects of pH and culture medium, slightly significant effects of fungal species, and highly significant interactions between all factors, including the three-way interaction medium, pH and fungal species. *T. arenaria* exhibited strong growth across all pH levels on PDA but showed marked inhibition on MEA at pH ≥ 6.0. In contrast, *T. pinoyi* responded more favorably to PDA than MEA at higher pH levels. For mycelial biomass production, no significant effect of species, pH, or medium was found when considered individually. However, a highly significant interaction was detected between species and solid-state culture for both wet- and dry-weight mycelium biomass production. *T. arenaria* has produced a greater biomass on MEA, while *T. pinoyi* performed better on PDA. In contrast, pH has shown no significant effect within the tested conditions. Inoculated *H. canariense* roots exhibited abundant fungal hyphae, vesicles, and endospores, together with a fungal mantle and Hartig net, confirming the establishment of a dual ectomycorrhizal–endomycorrhizal symbiosis. Overall, the results highlight strong species-specific responses and demonstrate that successful desert truffle cultivation requires optimization of culture medium and pH according to fungal species.

## 1. Introduction

Desert truffles are an important group of hypogeous fungi naturally adapted to arid and semi-arid environments, particularly in the Mediterranean region, North Africa, and the Middle East. Their development is closely linked to seasonal climatic conditions, soil characteristics, and symbiotic interactions with host plants, especially species of the genus *Helianthemum*. Beyond their ecological role in plant fungus interactions and adaptation to water-limited environments, desert truffles are highly valued as edible fungi because of their nutritional, socioeconomic, and potential bioactive properties. Their natural production, however, is irregular and strongly dependent on environmental conditions, making the development of controlled cultivation approaches increasingly important. A better understanding of desert truffle diversity, mycelial physiology, nutritional requirements, responses to culture media and pH, and ability to establish mycorrhizal associations with suitable host plants is therefore essential for improving their in vitro propagation and developing reliable cultivation strategies. Desert ecosystems display a species variety of desert truffles [1,2] which belong to four genera including *Delastria*, *Picoa*, *Terfezia*, and *Tirmania*. Nine species were recorded, *Delastria rosea*, *Picoa carthusiana*, *Picoa juniperi*, *Terfezia arenaria*, *Terfezia boudieri*, *Terfezia claveryi*, *Terfezia leptoderma*, *Tirmania pinoyi*, and *Tirmania nivea*. Desert truffles are mycorrhizal fungi belonging to the Ascomycota phylum, characterized by the production of hypogeous edible ascocarps. The genera *Terfezia* and *Tirmania* establish mycorrhizal associations with host plants, primarily with *Helianthemum* species, in arid and semi-arid ecosystems [3,4]. The genera *Terfezia* and *Tirmania* are collectively designated «desert truffles» given that they are predominantly discovered in arid and semi-arid Mediterranean ecosystems [5,6]. *Terfezia* species are capable of establishing diverse mycorrhizal associations, encompassing the formation of ectomycorrhiza, endomycorrhiza, and ect-endomycorrhiza, under specific environmental conditions [7]. *Terfezia arenaria* establishes a mycorrhizal association with the host plants, *Helianthemum sessiliflorum* and *Helianthemum guttatum*, as well as with other species of the *Cistaceae* family [8,9]. *T. arenaria* and *T. boudieri* (Pezizaceae) are considered the most prevalent species found in arid environments, and are of significant socioeconomic importance. Desert truffles are consumed in numerous countries across the Mediterranean Basin and the Persian Gulf and are utilized in traditional medicine, as well as being a highly esteemed food ingredient in various cuisines [10,11,12]. Desert truffles contain high levels of minerals (magnesium, calcium, iron), amino acids, protein, fiber, and carbohydrates, as well as fatty acids. Moreover, they are a rich source of compounds that possess antibacterial, anticancer, and antioxidant properties, which contribute to human health [9,11,13]. *Tirmania pinoyi* was shown to display antibacterial, anticancer, and antioxidant properties [14]. The cultivation of desert truffles represents a significant challenge. The successful co-culture of both the fungal symbiont and the host plant in sterile or semi-sterile conditions represents a crucial step. In addition, the lack of suitable inoculum sources has hampered the process. In 1970, the first in vitro germination of *Terfezia* ascospores was documented [15]. Since then, numerous formulations have been evaluated. For instance, Awameh et al. [16] utilized the medium developed at the Kuwait Institute for Scientific Research to isolate, preserve, and multiply various *Terfezia* species. However, the isolation of strains from *Terfezia* spp remained a significant challenge, as mycelial growth rates displayed considerable variability across different species and exhibited slow growth [17,18]. Moreover, most isolates exhibited an inability to grow before subculturing. A number of studies have been undertaken to isolate truffle mycelium from the gleba (ascocarps) or germinate ascospores. These studies include investigations led by Roth-Bejerano et al. [19]; Morte et al. [20]; Awameh and Alsheikh [16,21]; and Fortas and Chevalier [22]. Nevertheless, a paucity of publications concerning the obtention and isolation of pure cultures of these fungi (*Terfezia* and *Tirmania*) appears to be attributable in particular to low ascospore germination rates and low mycelium growth rates with different latency periods. These attributes enhance truffle’s significance as a crucial nutritional element, especially for nomadic populations inhabiting arid areas, where they serve as an indispensable source of nourishment for an extended, specific seasonal duration [23]. The current study aimed to isolate mycelial cultures from ascocarp fragments and characterize the microscopic features of two desert truffles, *Terfezia arenaria* (Moris) Trappe and *Tirmania pinoyi* (Maire) Malençon. The effects of pH (4.55, 5.62, 6.0, and 6.5), culture medium (PDA and MEA), and fungal species on apical growth rate (APG) and wet and dry mycelial biomass production (WWMBP and DWMBP) were evaluated at 28 °C. The study sought to identify species-specific responses and optimize in vitro cultivation conditions. In addition, the effectiveness of inoculating *Helianthemum canariense* seedlings with desert truffle mycelium and the establishment of mycorrhizal symbiosis was assessed through root colonization and associated fungal structures.

## 2. Materials and Methods

### 2.1. Collection of the Fungal Species

**Figure 1:**
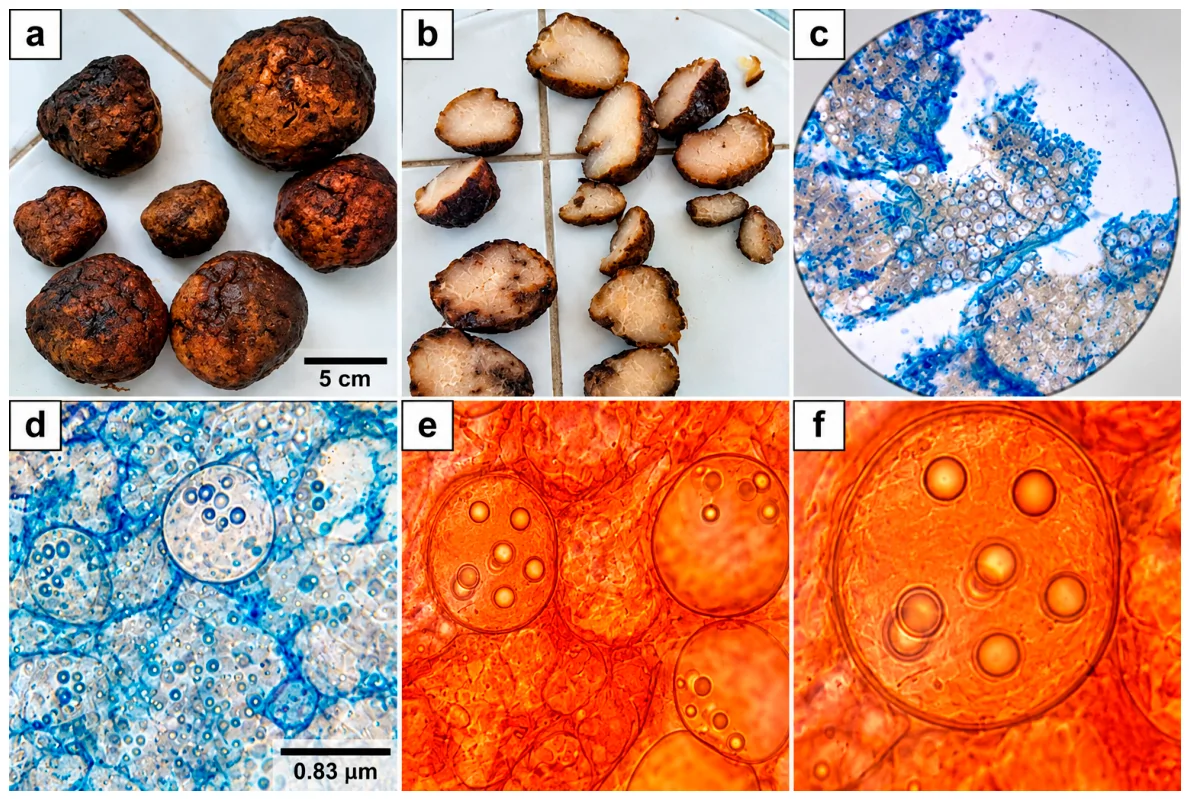
Macroscopic and microscopic characteristics of *Terfezia arenaria*. (**a**) Ascocarp; (**b**) longitudinal section of the ascocarp showing the gleba and peridium; (**c**) asci observed at ×100 magnification after treatment with NaOH(Sigma-Aldrich, St. Louis, MO, USA) and staining with Trypan Blue; (**d**) eight-spored ascus and free ascospores observed at ×400 magnification after treatment with NaOH and staining with Trypan Blue; (**e**) asci observed at ×400 magnification after treatment with KOH (Merck, Darmstadt, Germany) and staining with Congo Red; and (**f**) eight-spored ascus and free ascospores observed at ×1000 magnification after treatment with KOH and staining with Congo Red.

In February 2023, mature desert truffle ascocarps of *T*. *arenaria* (Moris) Trappe were harvested on limestone soil from the Oualidia region (32.685890, −8.982483), adjacent to their natural host plant, *Helianthemum salicifolium* and *Helianthemum guttatum*. In the same period, mature ascocarps of *T*. *pinoyi* (Maire) Malençon were collected in the Boujdour region (26.136805, −14.449187) in southern Morocco, where they were associated with *Helianthemum lippi*. The ascocarps were stored in paper bags in a fridge (4 °C) for 24 h before their use the following day. Figure 1a,b shows the ascocarps and sections (Gleba and Peridium) of *T*. *arenaria* and *T*. *pinoyi* respectively.

### 2.2. Molecular Identification of Desert Truffle Isolates Using PCR Sequencing

DNA was extracted from 200 mg of ascocarps tissue using the FastDNA SPIN Kit for Soil (MP Biomedicals, Illkirch, France) according to the manufacturer’s instructions. DNA purity was improved by adding 40 mg of polyvinylpolypyrrolidone (PVPP) during the initial extraction step, followed by an additional wash with 5.5 M guanidine thiocyanate prior to the use of the SEWS-M wash buffer. All DNA extractions were performed in duplicate and the samples were stored at −20 °C for further analyses. Amplification of the internal transcribed spacer (ITS) region of rDNA was performed for fungal communities associated with *Terfezia* and *Tirmania* ascocarps. All amplification products were analyzed using standard PCR protocols. The ITS region was amplified using the primers ITS1F (5′-CTTGGTCATTTAGAGGAAGTAA-3′) targeting the 18S rRNA gene, and ITS4 (5′-TCCTCCGCTTATTGATATGC-3′) targeting the 28S rRNA gene. Primer characteristics are summarized in Table 1.

PCR products were purified and subsequently sequenced by Eurofins Genomics (Ebersberg, Germany). Raw sequences were quality-checked, trimmed, and assembled using Codon Code Aligner v.10.0.3 (www.codoncode.com, accessed on 1 October 2026). Sequence identification was performed by comparing the assembled sequences against the NCBI nucleotide database using the BLASTn algorithm, and species assignment was based on the highest sequence similarity and coverage.

### 2.3. Microscopic Features of Terfezia arenaria and Tirmania pinoyi

A binocular loupe was employed to facilitate a detailed morphological examination of *T*. *arenaria* and *T*. *pinoyi* truffles, encompassing their macroscopic characteristics, particularly shape and color. This entailed an in-depth observation of key structures such as the ascocarps, ascus, peridium, and gleba. Morphological characterizations of *T*. *arenaria* and *T*. *pinoyi* were based on available keys, as referenced in the literature [24]. Fresh samples of *T*. *arenaria* and *T*. *pinoyi* were manually cut with a sharp blade, placed in a solution of 5% KOH or 5% NaOH, and heated at 90 °C for 10 min. They were colored using Trypan Blue, Fuchsin, eosin and Congo Red and then heated at 90 °C for 10 min. Magnification observations were made at ×400 for the sample shape, the number of ascospores per ascus, and their length and width under a light microscope. Dimensions of ascocarps, asci, mycelia, and ascospores were measured using Image J software (version 1.54u8, National Institutes of Health, Bethesda, MD, USA) and scanning electron microscopy.

### 2.4. Mycelial Cultures Isolated from Ascocarp Fragments on Solid-State Fermentation

Two types of truffle bodies were used, *T*. *arenaria* and *T*. *pinoyi*, as biological starting materials, based on their external color. Ascocarps were meticulously washed with tap water and subsequently surface-sterilized using 70% methanol. In vitro cultures were established from ascocarp fragments. Fungal isolation was performed by excising fresh tissue from the internal gleba. Under sterile conditions in a laminar flow hood, ascocarps were halved, and a small portion of the gleba was carefully excised with a sterile scalpel and directly transferred onto the culture medium. These fragments served as starting points for fungal isolation and culture. The resulting cultures were then subcultured. The incubation was conducted in the dark at 28 °C with different pH levels. Two culture media were used, PDA and MEA, and 15 g of agar was added per liter of the MEA medium to solidify it. Both culture media were autoclaved at 121 °C for 15 min. The cultures were maintained at 28 °C in Petri dishes containing agar media, at pH 4.55, pH5.62, pH 6 and 6.5 for both PDA and MEA solid-state fermentation [25].

### 2.5. Species Reactivation

Reactivation methods can enhance culture techniques and result in more efficient production of fungal mycelium [26]. A sample of PDA and MEA medium with mycelium was collected and deposited centrally in Petri dishes pre-pulled with MEA and PDA and incubated at 28 °C for 4 to 5 days [27].

### 2.6. Apical Growth Rate Measurement on PDA and MEA

The apical growth (APG) of the mycelial cultures was monitored by recording the time required for the mycelium to completely colonize the Petri dish. The apical growth rate (APG) of the mycelial culture was determined by subtracting the initial radius of the ascocarp fragment used as inoculum from the final radius measured at the end of the incubation period, and dividing the resulting radial extension by the incubation time: APG = (R_2_ − R_1_)/t. Where R_1_ is the initial radius of the ascocarp fragment inoculum, R_2_ is the final radius of the mycelial colony, and t is the incubation time (hours). The APG was estimated after incubation at 28 °C and expressed as cm h^−1^.

### 2.7. Measurement of Wet- and Dry-Weight Mycelia Biomass Production in Solid-State Fermentation

The dry weight of mycelia biomass (DWMB) was directly estimated using 250 mL Erlenmeyer flasks at a ratio of 100 mL per flask, which was subsequently filled with 100 mL of the suitable culture medium, either PDA or MEA, and autoclaved at 121 °C 15 min. A frozen inoculum square of *T*. *arenaria* and *T*. *pinoyi*, which contains mycelium, was placed at the center of each Erlenmeyer flask, which was subjected to incubation at a temperature of 28 °C at pH 6 and pH 6.5 for 30 days within the stove. Three replicates were set up for each test. After the incubation, the contents of Erlenmeyer flasks were delicately scraped using a pair of tweezers to evenly distribute the generated biomass, which was subsequently measured using a precise weighing scale.

### 2.8. Mycelial Inoculation and Microscopic Assessment of Mycorrhizal Colonization in Helianthemum canariense Seedlings

To further evaluate the efficacy of the developed mycelial culture, *Helianthemum canariense* seedlings were inoculated using a mycelial suspension method to rapidly establish symbiotic associations with *Terfezia* and *Tirmania* under controlled conditions. This procedure begins with the production of active mycelial biomass on solid media, which was then homogenized in sterile water using a shaker or blender to generate a suspension rich in viable hyphal fragments. The resulting suspension was applied directly to the root system of young seedlings at a rate of 20 mL per plant, ensuring intimate contact between the mycelium and roots to promote successful colonization. Root fragments were observed under a light microscope after clearing with 10% KOH and staining with 0.05% Trypan Blue. Endomycorrhizal structures were visualized by mounting the root fragments between a slide and cover slip. Additionally, the root system of *Helianthemum* seedlings was examined under a binocular microscope for the presence of ectomycorrhizal tips, the fungal mantle, and the formation of ectomycorrhizal morphotypes.

### 2.9. Statistical Analysis

The experimental data were analyzed using the statistical software STATISTICA version 12 (StatSoft Inc., Tulsa, OK, USA and were analyzed for statistical significance with a *p*-value lower than 0.05. The data were represented as the mean and the standard deviation. The present study aims to conduct a statistical analysis of the apical growth rate APG and the dry white mycelium biomass production DWMBP of *T*. *arenaria* and *T*. *pinoyi* grown on PDA and MEA solid media using a Factorial ANOVA test to make comparisons.

## 3. Results and Discussion

### 3.1. Taxonomic Assignment Based on DNA Sequencing Analysis

The molecular analysis carried out after sequencing the PCR products enabled precise identification of the studied isolates. The obtained sequences were compared with reference databases (such as GenBank) using alignment tools, revealing a high percentage of similarity with desert truffle species. The results allowed the sequences to be assigned to *Terfezia arenaria*, GenBank accession number (PZ742179.1) and *Tirmania pinoyi* GenBank accession number (PZ742216.1). This identification confirms the presence of these two taxa in the analyzed samples and validates the molecular approach as a reliable tool for the taxonomic characterization of ectomycorrhizal fungi. This is consistent with recent ITS/LSU-based phylogenetic work on North African *Tirmania*, in which *T. pinoyi* forms a distinct, well-supported lineage among four species [28], and with the ITS/BLAST 2.17.0-based identification of Algerian *Tirmania* material [29].

### 3.2. Microscopic Features of Terfezia arenaria

Morphological structures of *T*. *arenaria* illustrated in Figure 1a–f. The ascocarps of *T*. *arenaria* have a mean length of 8 cm and a sub-globose-to-lobed shape. The peridium was about 0.34 cm thick, ranging from light brown to dark brown, and had a smooth texture. On the other hand, the gleba was whitish to red-brown. Pale sterile veins separate the brown fertile pockets at maturity. The ascus length measured 80 μm and 65 μm in width and had eight ascospores. The ascospores were globose to sub-globose and hyaline, with a length of 18 μm including ornamentation. They were initially hyaline or yellow and became dark brown at maturity. The microscopic features of *T*. *arenaria* mycelia cultivated on solid-state culture and observed from ascocarps revealed that they were septate. The mycelium observed from ascocarps showed a mean diameter of 17.54 μm and 118.898 μm mean distance between two septa. The average hyphal diameter (17.54 μm) and septal spacing (118.898 μm) in *T. arenaria* were consistent with thick, resistant structures observed in other hypogeous fungi such as *Tuber*, where vesicles and anastomoses play critical roles in survival and vegetative persistence [30,31]. These features also suggest a potential symbiotic adaptation to stressful soil environments.

### 3.3. Microscopic Features of Tirmania pinoyi

**Figure 2:**
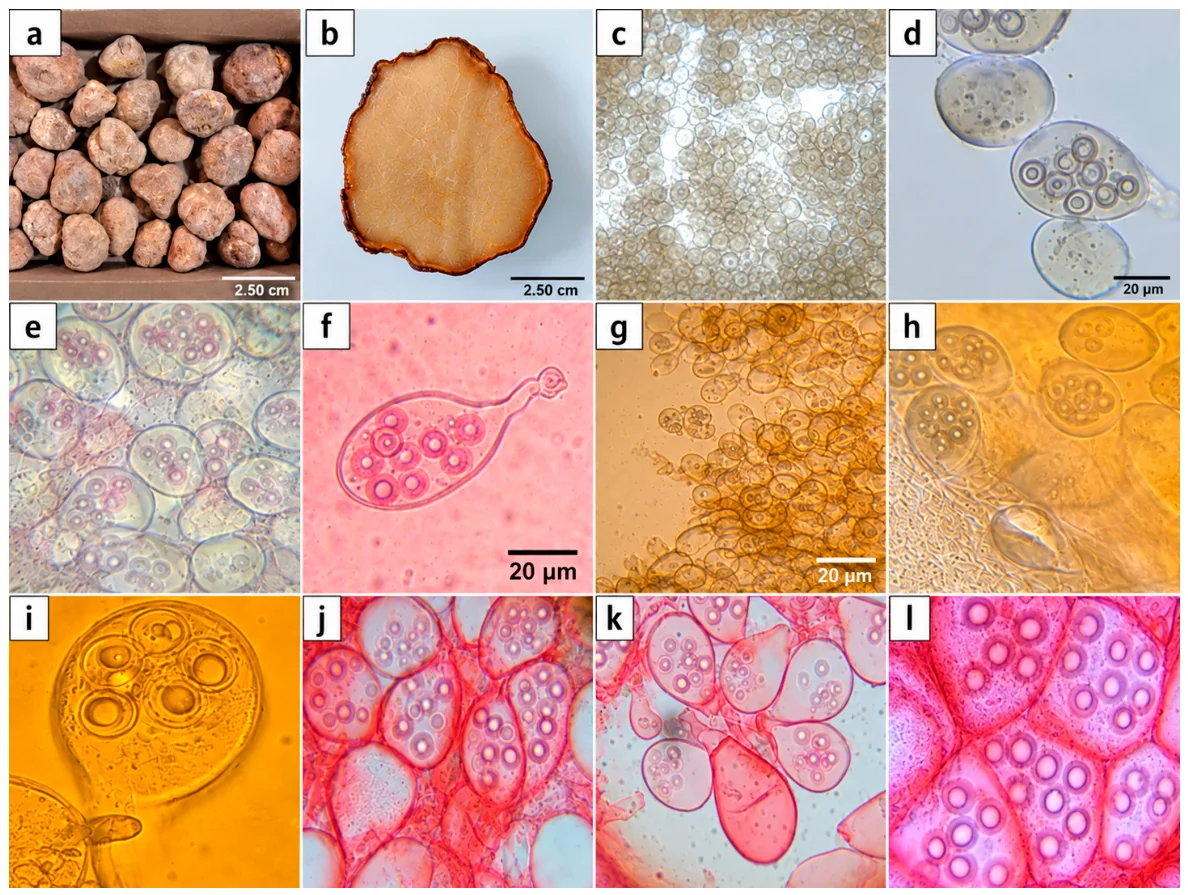
Macroscopic and microscopic characteristics of *Tirmania pinoyi*. (**a**) Ascocarp; (**b**) longitudinal section of the ascocarp showing the gleba and peridium; (**c**,**d**) asci and chains of asci after treatment with NaOH and staining with Trypan Blue; (**e**,**f**) asci and free ascospores, including eight-spored asci, after treatment with NaOH and staining with Fuchsin; (**g**–**i**) asci, chains of asci, and free ascospores after treatment with KOH and staining with eosin; and (**j**–**l**) asci, chains of asci, and ascospores after treatment with KOH and staining with Congo Red. Microscopic observations (**c**–**l**) were performed at ×400 magnification.

Morphological structures of *T*. *pinoyi* are illustrated in Figure 2a–l. *T. pinoyi* ascocarps usually have a sub-globose, pyriform shape and were 12 cm length on average. Their color can range from white to creamy, and their surfaces can be smooth, wrinkled, or cracked. The external envelope was formed by the *T*. *pinoyi* peridium. It was about 0.342 cm thick and pale yellowish-white to light brown. The *T*. *pinoyi* gleba flesh was white and fleshy, with small veins (furrows) and pale veins (furrows), pale yellowish, and solid. A microscopic analysis of the gleba of *T*. *pinoyi* revealed numerous mycelia, each containing ascospores. The ascus of *T. pinoyi* exhibits a mean length of 100 µm and a mean width of 80 µm with the pedicellate, and 36.888 µm in length and 26.102 µm in width without it. It is important to note that a unique ascus of *T*. *pinoyi* visualized under a microscope revealed that it was 55.644 µm in length with the pedicellate and 36.888 µm in length without it with a width of 26.102 µm. These structures were ellipsoidal to ovoid in form and contained eight ascospores; they possessed thin walls and short pedicels with a length of 18.503 µm and width of 4.684 µm Figure 2f. The ascospores of *T*. *pinoyi* were distributed in a free arrangement within the asci or outside of it and were ellipsoidal, with a mean length of 20 µm and a mean width of 14 µm The microscopic features of *T*. *pinoyi* mycelia grown on solid media revealed that they were septate. Septate, branched hyphae have likewise been described in *Terfezia claveryi* mycelium obtained from germinated ascospores [32]. This study reinforces the previously reported challenges in isolating and cultivating desert truffles, particularly those of the *Terfezia genus*, as highlighted by Fortas and Chevalier [22]. Nevertheless, the successful isolation and cultivation of *T. arenaria* and *T. pinoyi* from ascocarp fragments were consistent with the pioneering observations of Awameh and Alsheikh [16,21], who reported the first successful germination of ascospores from several desert truffle species. Cultivation on solid-state culture (PDA and MEA at 28 °C and pH 6–6.5) enabled the development of septate mycelia for both species [33].

### 3.4. Isolation of Mycelial Cultures and Apical Growth Rate from Ascocarp Fragments

**Figure 3:**
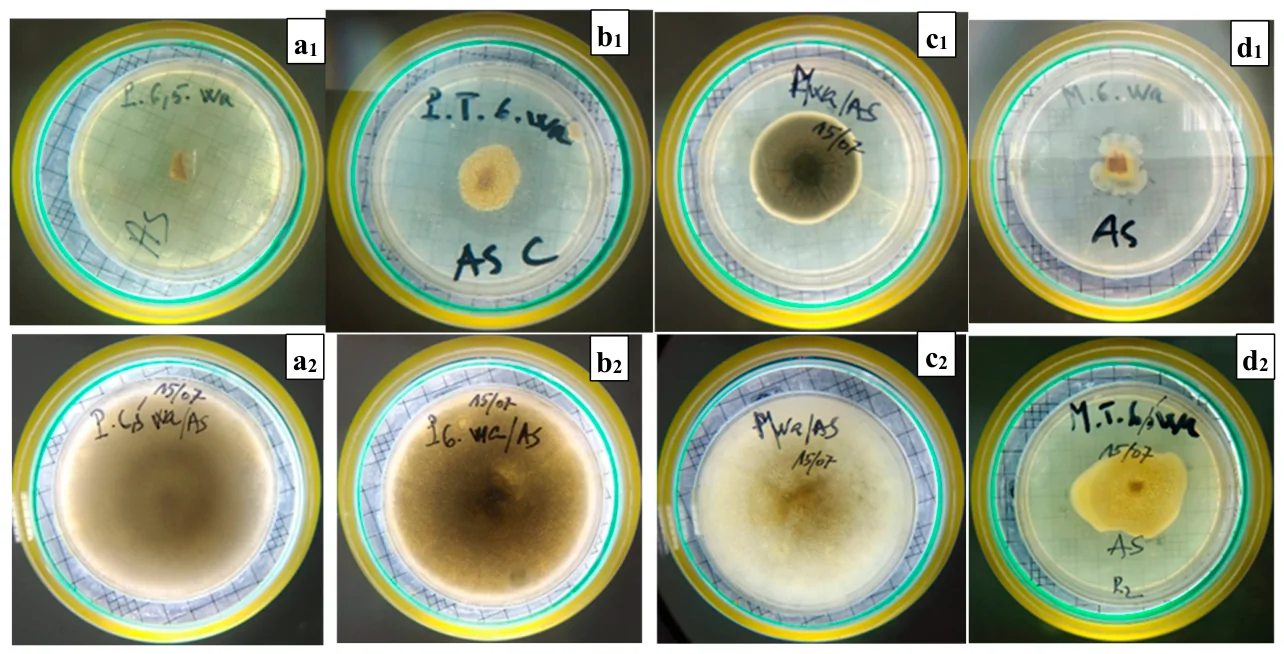
Macroscopic characteristics and mycelial growth of *Terfezia arenaria* cultured from ascocarp fragments on Potato Dextrose Agar (PDA) and Malt Extract Agar (MEA) under different pH conditions. (**a1**,**a2**) PDA at pH 6.5; (**b1**,**b2**) PDA at pH 6.0; (**c1**,**c2**) MEA at pH 6.5; and (**d1**,**d2**) MEA at pH 6.0. For each treatment, panels labeled “1” represent the initial stage following inoculation, whereas panels labeled “2” represent the final stage of mycelial growth.

**Figure 4:**
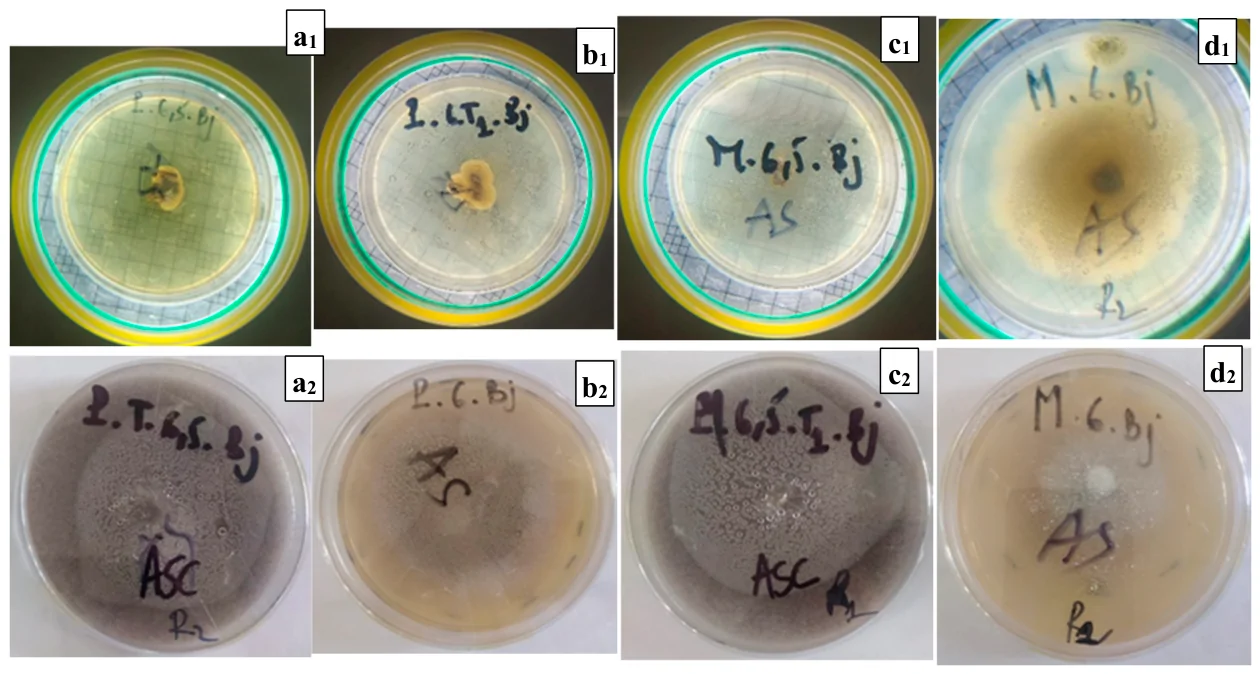
Macroscopic characteristics and mycelial growth of *Tirmania pinoyi* cultured from ascocarp fragments on Potato Dextrose Agar (PDA) and Malt Extract Agar (MEA) under different pH conditions. (**a1**,**a2**) PDA at pH 6.5; (**b1**,**b2**) PDA at pH 6.0; (**c1**,**c2**) MEA at pH 6.5; and (**d1**,**d2**) MEA at pH 6.0. For each treatment, panels labeled “1” represent the initial stage following inoculation, whereas panels labeled “2” represent the final stage of mycelial growth.

The results demonstrated that culturing fragments of the ascocarps resulted in the isolation of the mycelial structure on both PDA and MEA solid-state culture at 28 °C, pH values 6 and 6.5. Specifically, for *T*. *pinoyi* and *T*. *arenaria*, the utilization of PDA or MEA solid media facilitated a rapid development of the mycelial structure for both species, *T*. *arenaria* and *T*. *pinoyi* (Figure 3 and Figure 4). Fortas and Chevalier [22] subsequently investigated the ability of *Terfezia arenaria* spores to germinate in a variety of media, including the Kuwait Institute for Scientific Research medium composed of 10 g malt extract, 6.5 g peptone, 0.13 g KNO_3_, 0.55 g Ca (NO_3_), and 15 g agar; malt agar (MMA) was composed of 10 g malt extract, 15 g agar (Cristomalt 1%) and the INRA-Morizet medium. Other culture media were studied, in particular the Fontana medium and the modified Melin-Norkrans MMN medium, with the following composition: 10 g glucose, 0.05 g CaCl_2_, 0.025 g NaCl, 0.25 g (NH_4_)_2_HPO_4_, 0.5 g KH_2_PO_4_, 0.15 g MgSO_4_,7H_2_O, 0.012 g FeCl_3_, 0.0001 g thiamine, and 15 g agar. These were postulated to promote hyphal proliferation.

**Figure 5:**
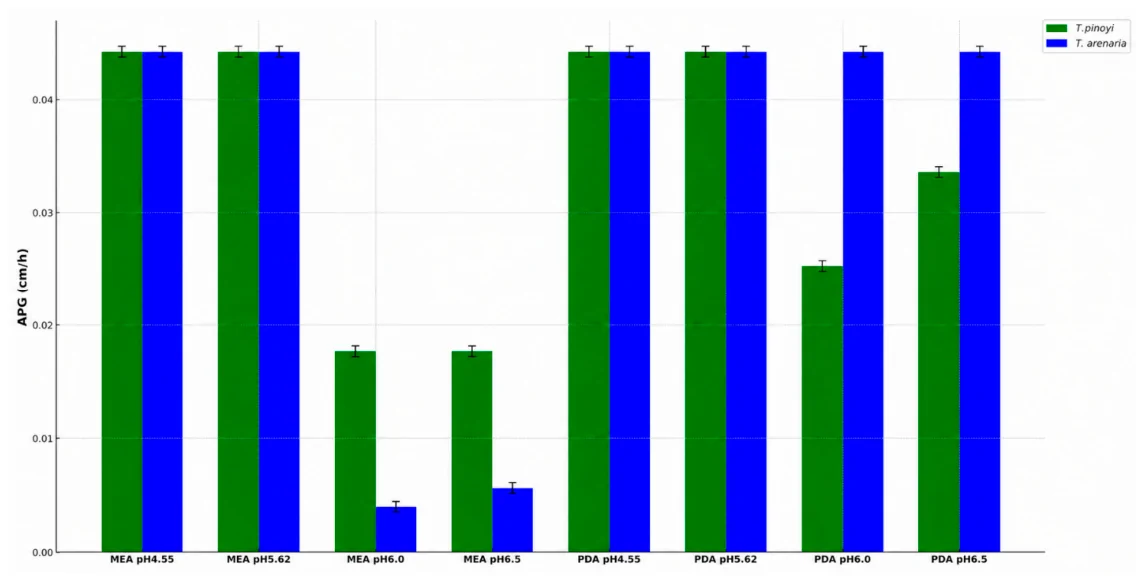
Apical growth rate of *T*. *arenaria* and *T*. *pinoyi* isolated from ascocarp fragments on PDA and MEA solid-state culture.

Univariate ANOVA revealed that all tested factors, culture medium, pH, and species, as well as their interactions, had statistically significant effects on apical growth rate APG. Both the culture medium (F = 3108.45, *p* < 0.000001) and pH (F = 2602.92, *p* < 0.000001) exhibited a strong influence on APG, while species had a weaker but still significant effect (F = 4.47, *p* = 0.042). The interactions between factors were also highly significant, including medium and pH (F = 1044.89), medium and species (F = 909.86), and pH and species (F = 10.23), all with *p*-values well below 0.001. Notably, the three-way interaction medium, pH and species were also significant (F = 322.98, *p* < 0.000001), suggesting that the combined response of fungal growth is dependent on the specific configuration of all three variables. The APG of *T. arenaria* and *T. pinoyi*, observed on PDA and MEA solid media, pH 4.55, and pH 5.62 at 28 °C, were 0.044 and 0.044 cm/h and 0.004 and 0.044 cm/h, respectively. Meanwhile, the APG of *T. arenaria*, observed on PDA and MEA solid media, at pH 6 and 6.5 at 28 °C, was 0.044 and 0.044 cm/h and 0.004 and 0.006 cm/h, respectively (Table 1 and Table S1). Therefore, highly significant pH levels had a major effect on growth when PDA and MEA media were used. The APG of *T*. *pinoyi*, observed on PDA and MEA solid media, at pH 6 and 6.5 at 28 °C, were (0.025; 0.034) cm/h and (0.018; 0.018) cm/h, respectively (Table S1), as shown in the Supplementary Material. Therefore, there was a significant effect of pH when PDA or MEA media were used (Table 2). For *T*. *pinoyi*, using PDA as a solid culture medium at pH 6.5 resulted in a 25% increase in APG compared with that observed at pH 6, while the same APG was observed in MEA solid medium. In addition, the APG observed at pH 6.5 on PDA solid medium was twice as large as that measured at pH 6 and 6.5 on MEA solid medium (Table 1, Figure 5). From a physiological perspective, APG analysis confirmed that all factors (solid-state culture, pH, and species) exert significant effects, with medium and pH having the most pronounced impact (F > 2600, *p* < 0.000001), in agreement with Morte et al. [20,34], who showed that *T. claveryi* was highly sensitive to growth-medium composition and pH. Notably, *T. arenaria* reached the highest APG (0.044 cm/h) on PDA at acidic pH levels (4.55 and 5.62), while *T*. *pinoyi* responded better to pH 6.5 on PDA, demonstrating a 25% increase in APG compared to pH 6 and double that observed on MEA. This highlights species-specific requirements and underlines the importance of the interaction between pH and solid-state culture. Similarly, a recent study on Moroccan *Terfezia boudieri* and *Tirmania nivea* reported a highly significant effect of the culture medium on apical growth rate, with PDA outperforming MEA, and PDA and MEA were also identified as optimal media for the mycelial growth of *Tuber koreanum* at pH 6.0 [35]. The inherently slow growth of desert truffle mycelia was consistent with observations by Kiraly and Bratek [36] for *T. terfezioides* and by Fontana [37], who noted that slow growth was a general characteristic of mycorrhizal fungi. Slow mycelial growth was also reported recently for *Terfezia claveryi* isolated from ascospores [32]. Nevertheless, the relatively higher APG of *T. arenaria* compared to *T. pinoyi* and to other *Tuber* species 0.01–0.03 cm/h on MMN [38] reflects a promising biological potential for cultivation optimization.

### 3.5. Productivity of Mycelial Biomass Production of Terfezia arenaria and Tirmania pinoyi in Solid-State Fermentation

**Figure 6:**
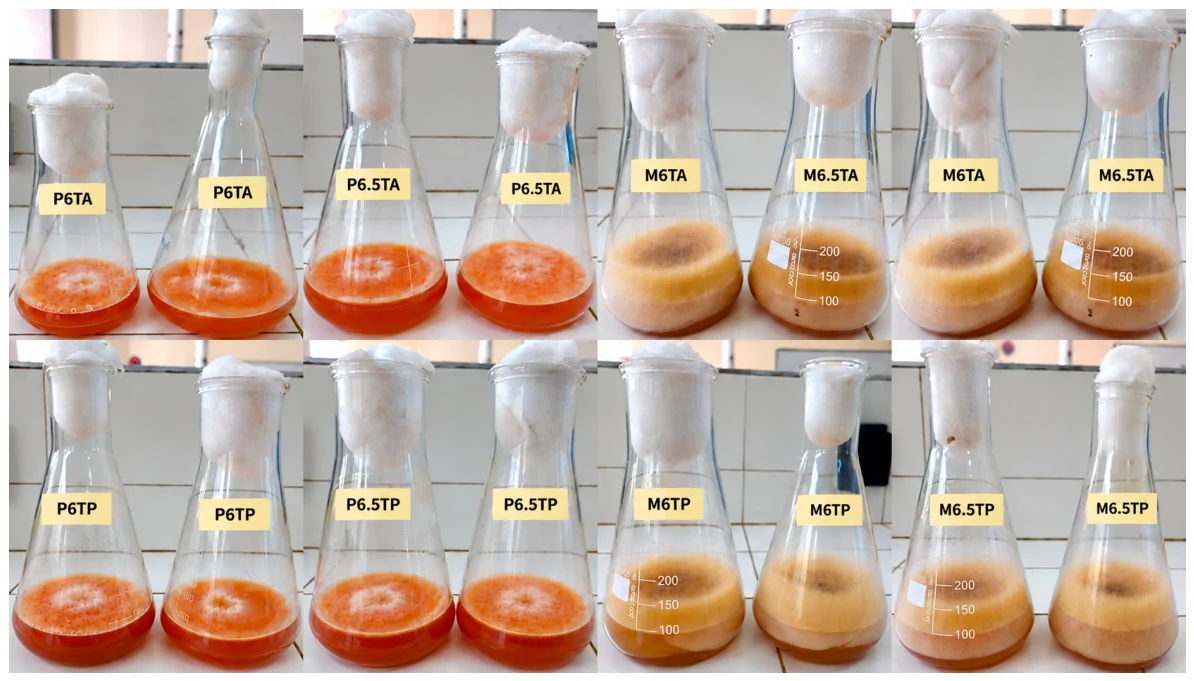
Wet-weight mycelial biomass production (WWMBP) of *T*. *arenaria* and *T*. *pinoyi* cultured on PDA and MEA solid media at pH 6 and 6.5 and 28 °C.

**Figure 7:**
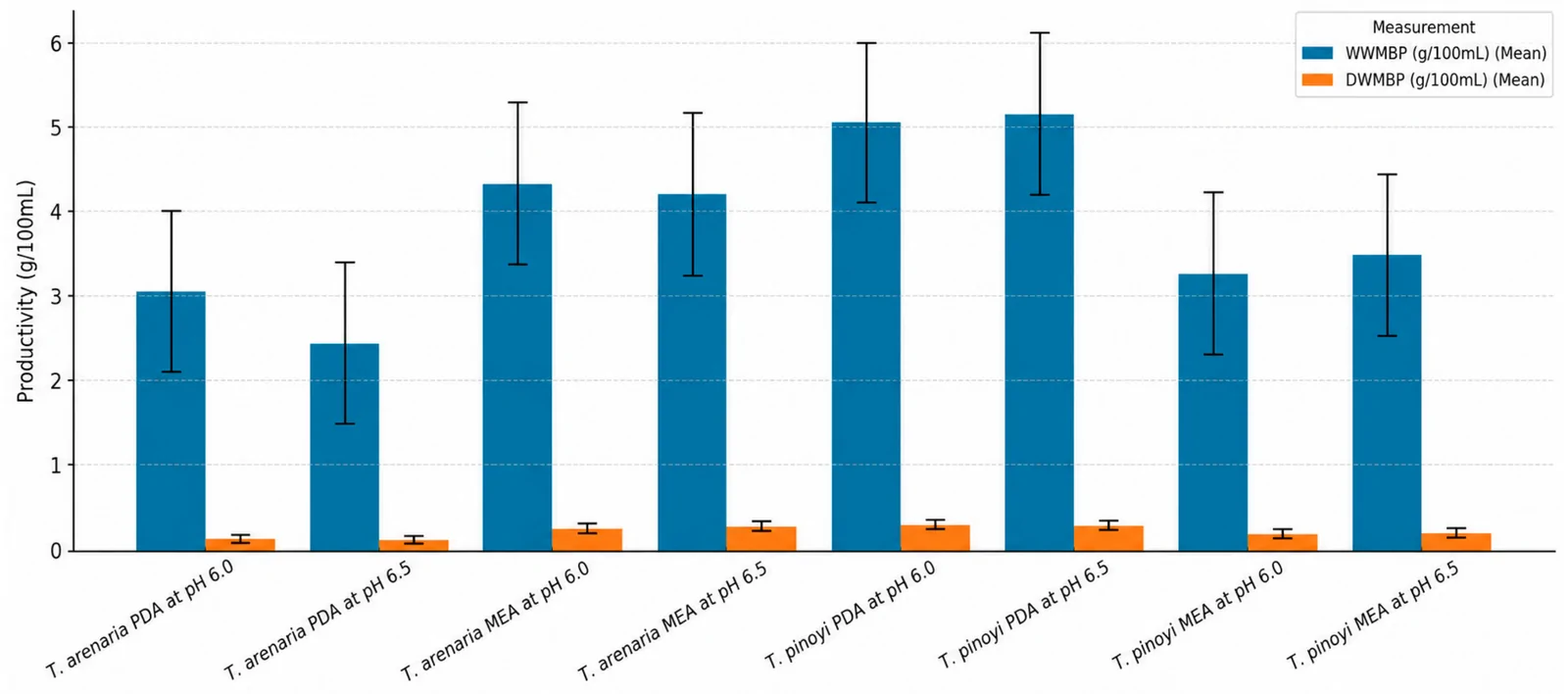
Productivity of wet-weight mycelial biomass (WWMBP) and dry-weight mycelial biomass (DWMBP) of *T*. *arenaria* and *T*. *pinoyi* isolated from ascocarp fragments and cultured on PDA and MEA solid media.

**Figure 8:**
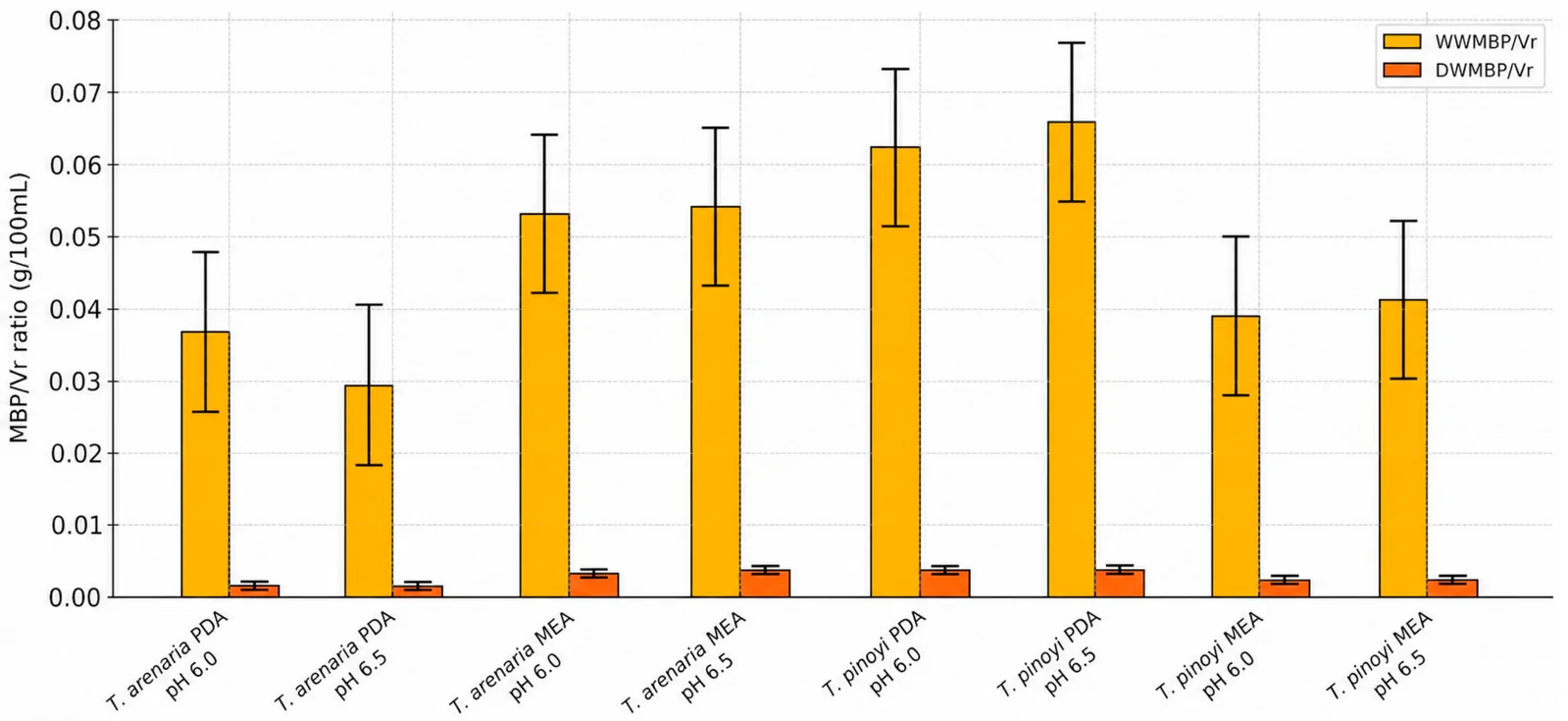
Productivity ratio of *T*. *arenaria* and *T*. *pinoyi* isolated from ascocarp fragments and cultured on PDA and MEA solid media.

Production of mycelial biomass of *T. arenaria* and *T. pinoyi* in solid-state culture ANOVA analysis revealed a highly significant interaction between fungal species and solid-state culture type for all measured variables, including wet weight of mycelial biomass production (WWMBP: F (1.8) = 5.85, *p* = 0.042), dry weight of mycelial biomass production (DWMBP: F (1.8) = 14.78, *p* = 0.0049), and their respective ratios normalized to the remaining volume. In contrast, the main effects of species, pH, or solid-state culture alone, as well as other interaction terms, were not statistically significant (*p* > 0.05). These findings suggest that biomass production response depends strongly on the species and medium combination, whereas pH had no significant influence under the tested conditions (Table 3, Table 4 and Table 5 and Figure 6 and Figure 7). We noted the contrast between the wet- and dry-weight mycelial biomass production of *T*. *arenaria* and *T*. *pinoyi* on two solid-state culture, PDA and MEA, at pH levels 6.0 and 6.5. Overall, *T*. *pinoyi* exhibited higher biomass production than *T*. *arenaria*, particularly on PDA solid-state culture, where the highest wet- and dry-weight mycelial biomass production values were recorded at pH 6.5 5.14 g/100 mL and 0.295 g/100 mL, respectively. For *T*. *arenaria*, MEA proved to be more favorable than PDA, with a slight increase in dry-weight mycelial biomass production from 0.26 to 0.29 g/100 mL as pH increased. In contrast, *T*. *pinoyi* had better production on PDA, with only minor variation between pH levels. The WWMBP/Vr and DWMBP/Vr ratios (expressing biomass per 100 mL of initial solid-state culture) support these findings, with *T*. *pinoyi* on PDA at pH 6.5 showing the highest efficiency (Figure 8). Although each condition was tested in three replicates (*n* = 3), clear trends emerged, indicating that *T*. *pinoyi* is generally more productive and that the choice of solid-state culture significantly influences mycelial biomass production for both species [39]. Mycelial biomass production analysis thus reveals a significant interaction between fungal species and solid-state culture type (wet biomass: *p* = 0.042; dry biomass: *p* = 0.0049), whereas individual effects of species, pH, or solid-state culture were not significant, which indicates that biomass production was more influenced by species-medium synergy than by pH alone. This is in line with the ecological plasticity reported by Navarro-Ródenas et al. [40,41], who showed that *T. claveryi* tolerates moderate water stress due to aquaporin gene (TcAQP1) expression. The current results also indicate that *T*. *pinoyi* consistently produces higher biomass on PDA, especially at pH 6.5 (up to 0.295 g/100 mL dry weight), whereas *T. arenaria* performs better on MEA, albeit at lower levels.

Despite the relatively low biomass compared to large-scale bioreactor yields reported for *T. claveryi* [42], where supplementation with vitamins, carbon (glucose), and nitrogen sources, along with pH buffering, increased biomass up to 3 g/L, this work provides a foundational understanding of cultivation parameters. Because desert truffle cultivation is increasingly viewed as a crop suited to arid and semi-arid zones [43], and in vitro mycelium can serve as inoculum for host plants, such baseline data are of practical relevance. That study also suggested that additional supplementation with essential growth factors such as cyclodextrins [44,45] could further enhance desert truffle biomass production in vitro.

### 3.6. Mycorrhizal Traits of Helianthemum canariense Seedlings

**Figure 9:**
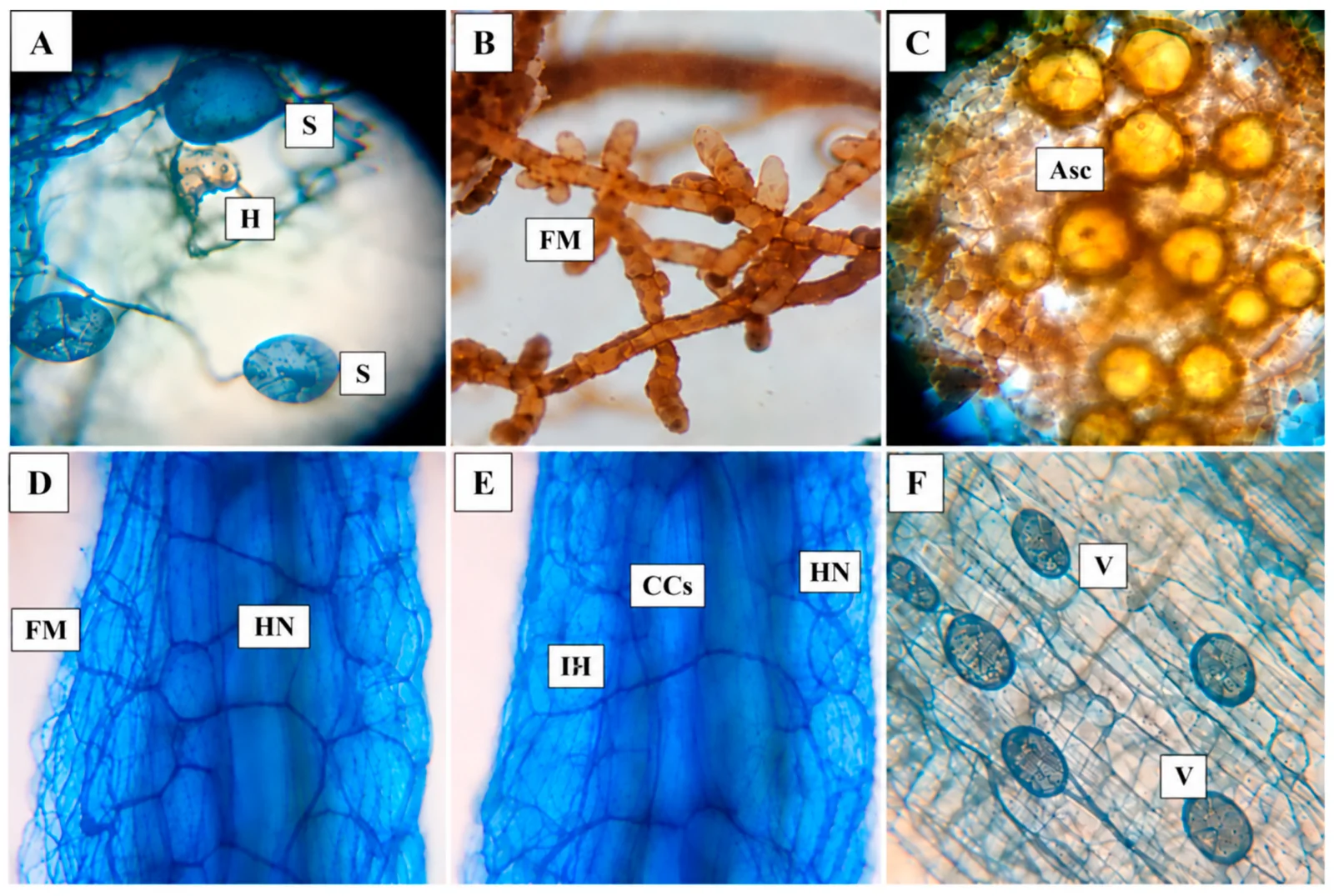
Mycorrhizal colonization and fungal structures observed in *Helianthemum canariense* seedlings following inoculation with *Terfezia arenaria* mycelium. (**A**) Endomycorrhizal colonization showing intracellular fungal structures within root cortical cells, including vesicles (V), hyphae (H), and spores (S). (**B**) Ectomycorrhizal colonization characterized by the formation of a well-developed fungal mantle (FM) surrounding the root tip. (**C**) Early formation of ascospores (Asc) in the rhizosphere of inoculated *H. canariense* seedlings. (**D**) Hartig net (HN). (**E**) Intracellular hyphae (IH) within cortical cells (CCs). (**F**) Vesicles (Vs) within root tissues.

The mycorrhizal traits of *Helianthemum canariense* roots were investigated to assess their endomycorrhizal and ectomycorrhizal status. Root samples were first cleared and stained to facilitate microscopic observation of fungal structures. The examination of root segments allowed for the detection of typical endomycorrhizal features like vesicles and chlamydospores (Figure 9A,F), and the number of ectomycorrhizal root tips was recorded to estimate the extent of colonization (Figure 9B); on average, 300 mycorrhizal short roots were recorded per plant. In addition, the presence of spores indicating the early formation of asci and ascospores at the soil surface was monitored as an indicator of active fungal development (Figure 9C). In addition, root observation showed a fungal mantle and Hartig net establishment (Figure 9D, E). These observations provide evidence of the establishment of mycorrhizal associations and contribute to understanding the symbiotic potential of *Helianthemum canariense* in relation to desert truffle species.

## 4. Conclusions

This study demonstrates that the successful in vitro cultivation of *T*. *arenaria* and *T*. *pinoyi* depends strongly on the interaction between species and culture media, while pH plays a significant role in modulating apical growth rates but not biomass production. The identification of optimal culture conditions is essential for improving biomass yields and for developing efficient inoculation systems for desert truffle cultivation. Future work should expand the pH range, increase replication, and explore the incorporation of key metabolic supplements to better mimic the fungi’s ecological niches and support industrial-scale production. The identification of optimal in vitro growth conditions provides a preliminary basis for developing reliable inoculum sources, which are essential for initiating desert truffle farming programs. Integrating pure culture techniques with host plant symbiosis, particularly with *Helianthemum* species, will be a critical next step to establish stable mycorrhizal associations under field conditions. These findings can ultimately contribute to scaling up inoculum production and advancing sustainable desert truffle cultivation as both an economic and ecological opportunity in arid regions.

## Figures and Tables

**Table 1:** Primer sequences and characteristics for ITS amplification.

Primer	Sequence (5′ → 3′)	Target Gene	Length (nt)	Mean Temperature (°C)	Amplicon (bp)	GC (%)
ITS1F	CTTGGTCATTTAGAGGAAGTAA	18S rRNA	22	58.0	633	36.36
ITS4	TCCTCCGCTTATTGATATGC	28S rRNA	20	61.0	633	45.00

**Table 2:** Univariate tests of significance for apical growth rate (cm/h), sigma-restricted parameterization, and effective hypothesis decomposition.

Effect	Sum of Squares	Degree. of Freedom	Mean Square	Fisher’s F Statistic	*p* Value
Intercept	0.0560	1	0.0560	88,658.78	0.0000
Culture media	0.0019	1	0.0020	3108.45	0.0000
pH	0.0049	3	0.0017	2602.92	0.0000
Species	0.0000	1	0.0000	4.47	0.0423
Culture media * pH	0.0019	3	0.0007	1044.89	0.0000
Culture media * species	0.0005	1	0.0006	909.86	0.0000
pH * species	0.0000	3	0.0000	10.23	0.000 1
Culture media * pH * species	0.0006	3	0.0002	322.98	0.0000
Error	0.0000	32	0.0000		

* interaction.

**Table 3:** Mean wet weights of mycelial biomass production (WWMBP), dry weights of mycelial biomass production (DWMBP), WWMBP/residual volume (Vr) and DWMBP/residual volume (Vr) per g/100 mL produced by *Terfezia arenaria* and *Tirmania pinoyi* at 28 °C, pH 6 and pH 6.5.

Species	Solid Media	pH	WWMBP (Mean ± Sd)	DWMBP (Mean ± Sd)	WWMBP/Vr (Mean ± Sd)	DWMBP/Vr (Mean ± Sd)	N
*T*. *arenaria*	PDA	6	3.0550 ± 0.9436	0.1250 ± 0.0469	0.0369 ± 0.0109	0.0015 ± 0.0006	3
*T*. *arenaria*	PDA	6.5	2.4450 ± 0.9436	0.1200 ± 0.0469	0.0295 ± 0.0109	0.0015 ± 0.0006	3
*T*. *arenaria*	MEA	6	4.3200 ± 0.9436	0.2600 ± 0.0469	0.0533 ± 0.0109	0.0032 ± 0.0006	3
*T*. *arenaria*	MEA	6.5	4.2000 ± 0.9436	0.2900 ± 0.0469	0.0543 ± 0.0109	0.0037 ± 0.0006	3
*T*. *pinoyi*	PDA	6	5.0550 ± 0.9436	0.3050 ± 0.0469	0.0625 ± 0.0109	0.0038 ± 0.0006	3
*T*. *pinoyi*	PDA	6.5	5.1400 ± 0.9436	0.2950 ± 0.0469	0.0660 ± 0.0109	0.0038 ± 0.0006	3
*T*. *pinoyi*	MEA	6	3.2750 ± 0.9436	0.1950 ± 0.0469	0.0392 ± 0.0109	0.0024 ± 0.0006	3
*T*. *pinoyi*	MEA	6.5	3.4850 ± 0.9436	0.2000 ± 0.0469	0.0414 ± 0.0109	0.0024 ± 0.0006	3

**Table 4:** Univariate results for each dependent variable, wet weight of mycelial biomass production (WWMBP), and dry weight of mycelial biomass production (DWMBP) per g/100 mL produced by *Terfezia arenaria* and *Tirmania pinoyi* at 28 °C, pH 6 and pH 6.5.

Effect	Degree. of (Freedom)	WWMBP (Sum of Squares)	WWMBP (Mean Squares)	WWMBP (Fisher F)	WWMBP (*p*-Value)	DWMBP (Sum of Squares)	DWMBP (Mean Squares)	DWMBP (Fisher F)	DWMBP (*p*-Value)
Intercept	1	239.8627	239.8627	134.7009	0.0000	0.8010	0.801025	182.0511	0.0000
Species	1	2.1536	2.1536	1.2094	0.3034	0.0100	0.010000	2.2727	0.1701
Solid media	1	0.0431	0.0431	0. 0242	0.8803	0.0025	0.0025	0.5682	0.4726
pH	1	0.0473	0.0473	0. 0266	0.8746	0.0001	0.0001	0.0227	0.8839
Species * Solid media	1	10.4168	10.4168	5.8498	0.0420	0.0650	0.0650	14.7784	0.0050
Species * pH	1	0.2627	0.2627	0.1475	0.7110	0.0002	0.0002	0.0511	0.8268
Solid media * pH	1	0.0946	0.0946	0.0531	0.8235	0.0006	0.0006	0.1420	0.7160
Species * Solid media * pH	1	0.0333	0.0333	0.0187	0.8946	0.0001	0.0001	0.0227	0.8839
Error	8	14.2457	1.7807			0.0352	0.0044		
Total	15	27.2968				0.1138			

* interaction

**Table 5:** Univariate results for each dependent variable, wet weight of mycelial biomass production (WWMBP)/residual volume (Vr) and dry weight of mycelial biomass production (WWMBP)DWMBP/residual volume (Vr) per g/100 mL produced by *T*. *arenaria* and *T*. *pinoyi* at 28 °C, pH 6 and pH 6.5.

Effect	Degree. of Freedom	WWMBP/Vr (Sum of Squares)	WWMBP/Vr (Mean Squares)	WWMBP/Vr (Fisher F)	WWMBP/Vr (*p*-Value)	DWMBP/Vr (Sum of Squares)	DWMBP/Vr (Mean Squares)	DWMBP/Vr (Fisher F)	DWMBP/Vr (*p*-Value)
Intercept	1	0.0367	0.0367	153.3265	0.0000	0.0001	0.0001	196.3574	0.0000
Species	1	0.0003	0.0003	1.2831	0.2901	0.0000	0.0000	2.1163	0.1838
Solid media	1	0.0000	0.0000	0.0504	0.8280	0.0000	0.0000	0.5499	0.4796
pH	1	0.0000	0.00000	0.0006	0.9806	0.0000	0.0000	0.1013	0.7584
Species * Solid media	1	0.0020	0.0020	8.3019	0.0204	0.0000	0.0000	18.5496	0.0026
Species * pH	1	0.0000	0.0000	0.1535	0.7055	0.0000	0.0000	0.0697	0.7984
Solid media * pH	1	0.0000	0.0000	0.0533	0.8232	0.0000	0.0000	0.1261	0.7317
Species * Solid media * pH	1	0.0000	0.0000	0.0979	0.7624	0.0000	0.0000	0.1278	0.7299
Error	8	0.0019	0.0002			0.0000	0.0000		
Total	15	0.0043				0.0000			

* interaction.

## Data Availability

The datasets generated and/or analyzed during the current study are provided in the manuscript.

## Supplementary Materials

The following supporting information can be downloaded at https://www.mdpi.com/article/10.3390/microorganisms14102394/s1: Table S1: effects of interaction among culture medium, pH and fungal species on mean apical growth rate (APG, cm/h).
