# Supplementary material for: Mycelial Production and Mycorrhizal Establishment of Terfezia arenaria and Tirmania pinoyi on Helianthemum canariense: A Morphological and Molecular Study
Source: Microorganisms. 2026 Oct 20;14(10):2394. doi: 10.3390/microorganisms14102394 (PMC13707509; doi:10.3390/microorganisms14102394)
Supplement: Supplementary file 1 [file microorganisms-14-02394-s001.zip › microorganisms-4584063-supplementary.pdf]

Supplementary Material

Table S1. effects of interaction among culture medium, pH and fungal species on mean apical growth rate (APG, cm/h).

Culture media	pH	Species	APG (cm/h) (Mean)	N
**MEA**	4.55	*T. pinoyi*	0.0443±0.0005	3
**MEA**	5.62	*T. pinoyi*	0.0443±0.0005	3
**MEA**	6	*T. pinoyi*	0.0177±0.0005	3
**MEA**	6.5	*T. pinoyi*	0.0177±0.0005	3
**PDA**	4.55	*T. pinoyi*	0.0443±0.0005	3
**PDA**	5.62	*T. pinoyi*	0.0443±0.0005	3
**PDA**	6	*T. pinoyi*	0.0253±0.0005	3
**PDA**	6.5	*T. pinoyi*	0.0340±0.0005	3
**MEA**	4.55	*T. arenaria*	0.0443±0.0005	3
**MEA**	5.62	*T. arenaria*	0.0443±0.0005	3
**MEA**	6	*T. arenaria*	0.0040±0.0005	3
**MEA**	6.5	*T. arenaria*	0.0057±0.0005	3
**PDA**	4.55	*T. arenaria*	0.0443±0.0005	3
**PDA**	5.62	*T. arenaria*	0.0443±0.0005	3
**PDA**	6	*T. arenaria*	0.0443±0.0005	3
**PDA**	6.5	*T. arenaria*	0.0443±0.0005	3
